# Apolipoprotein A-I Mimetic Peptide L-4F Suppresses Granulocytic-Myeloid-Derived Suppressor Cells in Mouse Pancreatic Cancer

**DOI:** 10.3389/fphar.2020.00576

**Published:** 2020-04-30

**Authors:** Meiyu Peng, Qi Zhang, Yanqing Liu, Xiangdong Guo, Jiyu Ju, Lingzhi Xu, Yuanyuan Gao, Daquan Chen, Dongzhen Mu, Rongxin Zhang

**Affiliations:** ^1^Department of Immunology, School of Basic Medical Sciences, Weifang Medical University, Weifang, China; ^2^Tianjin Key Laboratory of Acute Abdomen Disease Associated Organ Injury and ITCWM Repair, Institute of Acute Abdominal Diseases, Tianjin Nankai Hospital, Tianjin Medical University, Tianjin, China; ^3^Department of Breast Surgery, Yantai Yuhuangding Hospital, Yantai, China; ^4^Laboratory of Immunology and Inflammation, Department of Immunology, Key Laboratory of Immune Microenvironment and Diseases of Educational Ministry of China, Tianjin Key Laboratory of Cellular and Molecular Immunology, Key Laboratory of Hormones and Development (Ministry of Health), Tianjin Medical University, Tianjin, China; ^5^Department of Pharmaceutics, School of Pharmacy, Weifang Medical University, Weifang, China; ^6^School of Pharmacy, Yantai University, Yantai, China; ^7^Guangdong Province Key Laboratory for Biotechnology Drug Candidates, School of Life Sciences and Biopharmaceutics, Guangdong Pharmaceutical University, Guangzhou, China

**Keywords:** pancreatic cancer, L-4F, PMN-MDSCs, STAT3, anti-tumor

## Abstract

L-4F is an apolipoprotein A-I (ApoA-I) mimetic peptide, it was engineered to imitate the anti-inflammatory and anti-oxidative activity of ApoA-I. In this paper, H7 cell was used to construct a mouse model of pancreatic cancer in situ, and the mice were treated with L-4F. Then, the development of pancreatic cancer and myeloid-derived suppressor cells (MDSCs) infiltration were investigated *in vivo*. After L-4F treatment, the differentiation, proliferation and apoptosis of MDSCs were detected *in vitro*. Moreover, we test its effects on the immunosuppressive function of MDSCs ex vivo. The results show that L-4F significantly reduced the tumorigenicity of H7 cells. L-4F suppressed granulocytic myeloid-derived suppressor cells (PMN-MDSCs) differentiation and inhibited the accumulation of PMN-MDSCs in the mouse spleen and tumor tissue. L-4F weakened the immunosuppressive function of MDSCs, resulting in decreased production of ROS and H_2_O_2_ by MDSCs, and increased T cell proliferation, interferon γ and tumor necrosis factor β secretion, and CD3^+^CD4^+^ T and CD3^+^CD8^+^ T cell infiltration into the mouse spleen and pancreatic cancer tissue. Furthermore, L-4F significantly down regulated the STAT3 signaling pathway in PMN-MDSCs. These results indicated that L-4F exerts an effective anti-tumor and immunomodulatory effect in pancreatic cancer by inhibiting PMN-MDSCs.

## Introduction

Myeloid-derived suppressor cells (MDSCs) include early bone marrow progenitors and precursors of granulocytes, macrophages, and dendritic cells (Gabrilovich and Nagaraj, 2009). In mice, MDSCs have been divided into two main categories: CD11b^+^Ly6C^+^Ly6G^−^ monocytic MDSC (Mo-MDSC) and CD11b^+^Ly6C^low/neg^Ly6G^+^ granulocytic MDSC (PMN-MDSC). In tumor-bearing mice, Mo-MDSC and PMN-MDSC is different in immunosuppressive function. Tumor-infiltrating MDSCs and residing in spleen and blood MDSCs have the same phenotypes. But, tumor-infiltrating MDSCs has higher suppressive function than blood or splenic MDSCs (Parker et al., 2015).

Healthy individuals (human and mouse) can found MDSCs at low levels in the peripheral blood. However, MDSCs levels increase under pathological conditions such as cancer (Almand et al., 2001), inflammation (Dorhoi and Kaufmann, 2015), and autoimmune disease (Wang et al., 2016). Increasing clinical evidence shows that the levels of circulating MDSCs in almost all patients with malignant tumors are elevated, and these levels may be directly related to the clinical cancer stage, metastatic tumor burden, and prognosis (Diaz-Montero et al., 2009; Gabitass et al., 2011; Eruslanov et al., 2012; Montero et al., 2012). Compared with healthy individuals, patients with pancreatic cancer demonstrate that the frequency of MDSCs in the bone marrow and peripheral circulation increased in patients with pancreatic cancer, which was associated with disease stage (Porembka et al., 2012). Targeting MDSCs, and combined with traditional immune-based therapies, it is possible to produce more effect and improve cancer treatment (Finke et al., 2011; Gabrilovich et al., 2012).

IL-6 could increase the infiltration of MDSCs, and it also can induce MDSCs to increase the production of IL-6 (Oh et al., 2013). Multiple signal transduction pathways, transcription factors, take part in the regulation of accumulation and function in MDSCs. One of these factors, MDSCs expansion, is mainly driven by STAT3 activation. In vivo, the receptor tyrosine kinase inhibitor sunitinib could decrease the accumulation of MDSCs by inhibiting STAT3 signaling pathway (Xin et al., 2009). STAT3 also take part in enhances MDSCs suppressive activity (Kujawski et al., 2008). The production of ROS by MDSCs is also regulated by STAT3, the activation of STAT3 increases ROS levels (Corzo et al., 2009).

Apolipoprotein A-I (ApoA-I), is one of the main protein components of high-density lipoprotein (HDL). ApoA-I possess anti-inflammatory and anti-oxidant properties. ApoA-I mimetic peptide 4F (L-4F), contains 18 amino acids and contains a Class A amphipathic helix with a polar and a nonpolar face that allows it to bind lipids. Similar to ApoA-I, L-4F retain the anti-inflammatory activity of ApoA-I (Xie et al., 2016). L-4F have shown positive effects when used to treat cancer or decrease inflammation (Gupta et al., 2005; Vaziri et al., 2009). Others studies have shown that L-4F plays effective anti-inflammatory properties. L-4F markedly decrease the serum Interleukin-6 (IL-6), tumor necrosis factor (TNF)-α, and Interleukin-1β (IL-1β) levels in obese mice (Peterson et al., 2008). L-4F suppresses the levels of TNF-α and IL-6, which secreted by LPS-stimulated neutrophils (Sharifov et al., 2014). L-4F inhibits LPS-induced inflammation by decreasing the production of cytokines, chemokines, and adhesion molecules (Gupta et al., 2005). Our recent study showed that L-4F treatment inhibited tumor progression significantly is relevant to reduce the secretion of IL-17A, IL-6, GM-CSF and IL-1β in the tumor tissue, and suppressed tumor-associated macrophage (TAM) differentiation and infiltration of the tumor tissue, and L-4F inhibited M2 macrophage differentiation, it was relate to the inhibition of STAT3 and MAPK pathways (Peng et al., 2017). However, the effect of L-4F on MDSCs is unclear. In this study, we aimed to investigate whether L-4F could inhibit the progression of pancreatic cancer by regulating MDSCs, and to determine the mechanism. Our results show that L-4F treatment significantly decreased the infiltration of PMN-MDSCs but not MO-MDSCs in mouse pancreatic cancer models. Furthermore, L-4F inhibited the differentiation of PMN-MDSCs and weakened the immunosuppressive function of PMN-MDSCs by decreasing the phosphorylation of STAT3.

## Materials and Methods

### Cell Lines

The highly metastatic mouse pancreatic cancer cell line H7 was kindly provided by professor Min Li (The Vivian L. Smith Department of Neurosurgery, Department of Integrative Biology & Pharmacology, The University of Texas Medical School at Houston). H7 cells was established using a method described by Wang et al. (2001) *in vivo*. H7 cells were cultured in Dulbecco’s modified Eagle’s medium (DMEM) containing 100 U/ml streptomycin, 100 U/ml penicillin (Gibco, USA), and 10% fetal bovine serum (FBS) in a humidified atmosphere of 5% CO2 at 37°C.

### Animals and the Tumor Model

Six to 8 weeks old female C57BL/6 mice were obtained from the Experimental Animal Center of Military Medical Sciences (Beijing, China), and acclimated for at least 1 week in the specific pathogen free cages before experimentation. All of the experiments were approved by the animal ethics committee of Weifang Medical University.

Anesthesia in mice, the abdomen of mice was disinfected, and made an approximate 1 cm longitudinal incision in the left upper abdomen. The tip of the pancreatic tail was grasped gently, and the pancreas and spleen were externalized in a lateral direction until fully exposed. H7 cells (1×10^6^ cells suspended in 50 μl of PBS) were injected into pancreas using 1 ml syringe with 27-gauge needle. Mice were randomly divided into L-4F treatment group (n=24) and Sc-4F control group (n=24) by daily intraperitoneal injection beginning on day 3. L-4F was synthesized from all L-amino acids, the peptide is Ac-D-W-F-K-A-F-Y-D-K-V-A-E-K-F-K-E-A-F-NH2. Sc-4F contained the same amino acids as the 4F peptide but arranged in the sequence Ac-D-W-F-A-K-D-Y-F-K-K-A-F-V-E-E-F-A-K-NH2. L-4F and Sc-4F synthesized by Lt Bio-Scientific Co.Ltd. The purity of these peptides is >95%. The vehicle was 50 mM ammonium bicarbonate containing 0.1 mg/ml Tween-20, pH=7.0. Mice were sacrificed after 14 d of treatment and the tumor tissue and spleen were collected for further study. Tumor tissue was weight.

### Isolation of Immunocytes From Spleen and Tumor Tissue

Spleen and tumor tissue were minced into small pieces, and single-cell suspensions were obtained by grinding these tissues and filtering them through a 40-μm cell strainer (BD Biosciences, USA). Immunocytes were isolated using Ficoll density gradient centrifugation.

### Flow Cytometric Analysis of the Infiltrating Immunocytes

The isolated immunocytes were blocked with purified rat anti-mouse CD16/32 antibodies (BD Biosciences, USA) for 30 min at 4°C, then stained with antibodies for 30 min at 4°C, washed and analyzed by a BD FACSVerse flow cytometer (BD Biosciences, USA). The following monoclonal anti-mouse antibodies were used: anti-CD11b-BV421, anti-LY6C-APC, anti-LY6G-PE, anti-CD3-PerCP, anti-CD4-APC-H7, and anti-CD8-FITC (BD Biosciences, USA). The labeling cells were assessed using a BD FACSVerse flow cytometer (BD Biosciences, USA). The acquired data were analyzed using FlowJo 7.6 software (TreeStar, Inc.).

### *In Vitro* Induction of MDSCs

Bone marrow cells were prepared as described by Wu et al. (2012). The method of MDSC induction using the bone marrow cells was performed according to a previously published paper (Marigo et al., 2010). Briefly, 4×10^6^ bone marrow cells per well were cultured in a 6-well plate in 2 ml of medium supplemented with 0.5 ng/ml recombinant mouse GM-CSF (Millipore, USA), in a 5% CO_2_ atmosphere for 4 d.

In addition, to detect the effect of L-4F on MDSC differentiation, Sc-4F/L-4F (20 μg/ml) was added in the process of inducing differentiation of MDSC on day 0.

The cell culture medium was DMEM supplemented with 2 mM L-glutamine, 20 μM 2-ME, penicillin (100 U/ml), streptomycin (0.1 mg/ml), and 10% heat-inactivated FBS.

### Cell Proliferation and Apoptosis Assay

As described above, mouse MDSCs were induced *in vitro*. Then induced MDSC cells were sorted using anti-mouse Ly-6G and Ly-6C particles-DM (BD Biosciences, USA). Ly6G^+^Ly6C^+^ cells (MDSC) proliferation and cell division was assayed by using carboxyfluorescein succinimidyl ester (CFSE) (Invitrogen, USA) labeling. Cell apoptosis was assessed using a MitoScreen (JC-1) assay kit (BD Biosciences, USA) according to the manufacturer’s protocol. The labeled cells were treated with vehicle or the different concentration of L-4F (5, 10, 20 μg/ml) for 48 h. Then, the cells were assessed using BD FACSVerse flow cytometer and quantified with FlowJo 7.6 software.

### Assays for the Suppression of T Cells by MDSCs

MDSCs were isolated from the spleen and tumor tissue from L-4F/Sc-4F treated mouse using anti-mouse Ly-6G and Ly-6C particles-DM (BD Biosciences, USA). The purity of each cell population was > 99%. The immunocytes from spleen (splenocytes) from C57BL/6 mice were isolated. A total of 2×10^5^/well splenocytes were stimulated with coated 6 µg/ml anti-CD3 (BD Biosciences, USA) and 6 µg/ml soluble anti-CD28 mAbs (BD Biosciences, USA) for 3 d and co-cultured at a 4:1 ratio with sorted MDSCs in 96-well flat-bottom plates. After 3 d, the cells were stained with anti-CD3-PerCP, anti-CD4-APC-H7, and anti-CD8-APC. Then the CD3^+^CD4^+^ and CD3^+^CD8^+^ lymphocytes was analyzed.

The culture supernatants were collected to detect the concentration of IFN-γ (interferon-γ) using an mouse IFN-γ ELISA assay kit (BD Biosciences, USA) and TNF-β using an mouse TNF-β ELISA assay kit (BioLegend, USA) according to the manufacturer’s protocol. Each experiment was performed in triplicate.

The culture medium consisted of RPMI 1640 medium supplemented with L-glutamine (2 mM), penicillin (100 U/ml), streptomycin (0.1 mg/ml), 2-ME (50 μ M), and 10% heat-inactivated FBS.

### Detection of ROS and H_2_O_2_ in MDSCs

MDSCs were isolated from the spleen and tumor tissue from L-4F/Sc-4F treated mouse using anti-mouse Ly-6G and Ly-6C particles-DM (BD Biosciences, USA). The purity of each cell population was > 99%. ROS activity was detected by using a fluorometric intracellular ROS kit (Sigma-Aldrich, USA) and the concentration of H_2_O_2_ was detected by using an Intracellular hydrogen peroxide assay kit (Sigma-Aldrich, USA) according to the manufacturer’s protocol. Mean fluorescence intensity (MFI) was analyzed using FlowJo 7.6 software (BD Biosciences, USA).

### Western Blotting Analysis

As described above, mouse MDSCs were induced *in vitro*. On the fourth day, the cells were incubated with L-4F at a concentration of 0, 0.1, and 0.25 μg/ml for 12 h, and then stimulated with concentration of 100 ng/ml recombinant mouse IL-6 for 15 min at 37°C. Finally, the total protein was extracted. Western blotting was performed to detect total and phosphorylated STAT3 (p-STAT3) and GAPDH in the mice bone marrow cells derived MDSCs. Briefly, protein concentration was determined by BCA protein quantification kit (Thermo, USA). Then, the proteins were electrophoresed by SDS-PAGE and transferred to polyvinylidene fluoride membranes. The membranes incubated with rabbit anti-mouse STAT3, p-STAT3 (PY705), and GAPDH antibody (Cell Signaling Technology, USA) overnight at 4°C depending on the target protein position. Subsequently, they were incubated with anti-rabbit IgG (Cell Signaling Technology, USA) and detected by chemiluminescence imaging system. The gray intensity of related proteins expression was analyzed by Quantity One.

### Flow Cytometric Analysis of Intracellular Staining

MDSCs were isolated from the spleen and tumor tissue from pancreatic cancer mouse model using anti-mouse Ly-6G and Ly-6C particles-DM (BD Biosciences, USA). Then, the MDSCs were incubated with L-4F at a concentration of 0, 0.1, and 0.25 μg/ml for 12 h, and then stimulated with 100 ng/ml recombinant mouse IL-6 (BD Bioscience, USA) for 15 min at 37°C. First, these cells were stained with anti-LY6C-APC and anti-LY6G-PE (BD Biosciences, USA). Second, these cells were fixed in a single step using BD Phosflow™ Lyse/Fix buffer for 10 min at 37°C. Third, these cells were permeabilized in BD Phosflow™ Perm Buffer III for 30 min on ice. Fourth, these cells were then stained with anti-p-STAT3 (PY705)-PE for 30 min at room temperature. Finally, the cells were assessed using a BD FACSVerse flow cytometer. The percentage of p-STAT3^+^ cells in PMN-MDSC was analyzed using FlowJo 7.6 software (BD Biosciences, USA).

### Statistical Analysis

Each experiment was performed in triplicate. All values were presented as the mean value ± the standard deviation (SD). Comparisons between two groups were performed using Student’s paired t-test, and one-way analysis of variance was used for comparisons the multiple groups as indicated. Statistical analysis was performed using SPSS 10.0 software.

## Results

### L-4F Inhibits the Accumulation of PMN-MDSCs in Mice With Pancreatic Cancer

**Figure 1:**
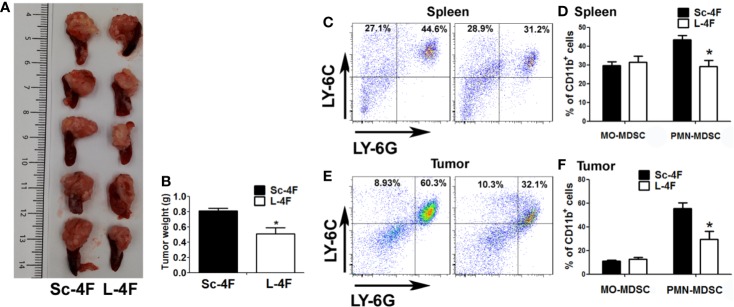
L-4F inhibits the infiltration of PMN-MDSCs in mice with pancreatic cancer. H7 cells were implanted into the pancreas. Mice were euthanized after 2 weeks of L-4F or Sc-4F treatment. **(A)** Representative tumors from Sc-4F- or L-4F-treated mice. **(B)** Final tumor weights (*P < 0.05). **(C, E)** One representative result from each experiment. **(D)** The percentages of MO-MDSCs and PMN-MDSCs among the splenocytes and **(F)** tumor-infiltrating cells in the tumor tissue (*P < 0.05).

Similar to our previous research (Peng et al., 2017), compared with the tumors in the Sc-4F-treated mice, the tumors in the L-4F-treated mice were significantly light (0.808 g vs 0.506 g, P < 0.05) (**Figures 1A, B**). Then we detected the infiltration of MDSCs in the spleen and tumor tissue by using flow cytometry. The populations of PMN-MDSCs and MO-MDSCs were assessed using the CD11b^+^Ly-6G^+^Ly-6C^Low^ and CD11b^+^Ly^−^6G^−^Ly-6C^+^ phenotypes as markers, respectively. Compared with Sc-4F-treated group, the percentages of PMN-MDSCs in the L-4F-treated group was significantly decreased in the spleen (43.33% *vs* 29.1%, respectively, P < 0.05) (**Figures 1C, D**) and tumor (55.32% *vs* 29.27%, respectively, P < 0.05) (**Figures 1E, F**). On the contrary, L-4F did not inhibit the increase in the accumulation of MO-MDSCs in the spleen (29.67% in the Sc-4F-treated group *vs* 31.33% in the L-4F-treated group, NS) (**Figures 1C, D**) or tumor (10.93% in the Sc-4F-treated group *vs* 12.74% in the L-4F-treated group, NS) (**Figures 1E, F**).

### L-4F Suppresses PMN-MDSCs Differentiation

**Figure 2:**
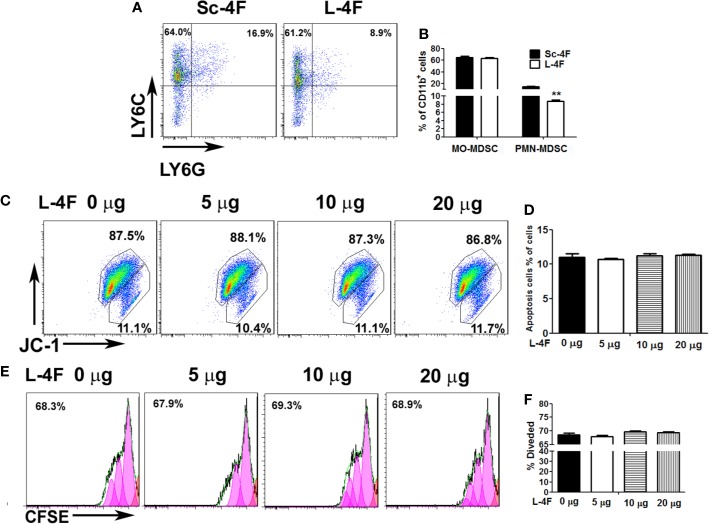
The effect of L-4F on MDSCs differentiation, proliferation and apoptosis. Bone marrow cells were treated with Sc-4F/L-4F (20 μg/ml) on day 0 of the MDSC induction. **(A)** One representative result from each experiment. **(B)** The percentages of MO-MDSCs and PMN-MDSCs among the induced cells (**P < 0.01). **(C, E)** MDSCs were treated with L-4F (0, 5, 10, or 20 μg/ml). One representative result from each experiment is shown. **(D)** The percentage of apoptotic cells and **(F)** the percentage of divided cells treated with L-4F at 48 h.

To further investigate the effect of L-4F on the differentiation of PMN-MDSCs *in vitro*, we induced mouse bone marrow-derived MDSCs using GM-CSF in the presence of Sc-4F or L-4F. As shown in the **Figures 2A, B**, the number of PMN-MDSCs was decreased significantly in L-4F-treated group with compared to the Sc-4F-treated group (8.74% *vs* 15%, respectively, P < 0.01). In contrast, there were no significant changes in the MO-MDSC populations (64.92% in the L-4F-treated group *vs* 63.32% in the Sc-4F-treated group, NS). These results indicate that L-4F can inhibit the differentiation of PMN-MDSCs populations *in vitro*.

### L-4F Did Not Reduce Proliferation or Induce Apoptosis of MDSCs

MDSCs were treated with different concentrations of L-4F (0, 5, 10, or 20 μg/ml). As shown in **Figures 2C, D**, the populations of apoptotic cells were not changed obviously with L-4F treatment compared with the untreated cells (11%, 10.73%, 11.23%, and 11.27%, for 0, 5, 10, and 20 μg/ml, respectively, NS). In addition, as shown in **Figures 2E, F**, the percentage of divided cells was not reduced obviously in the L-4F-treated group compared to the untreated or low dose-treated groups (68.63%, 68.02%, 69.72%, and 69.38% for 0, 5, 10, and 20 μg/ml, respectively, NS).

### L-4F Increases T Cell Infiltration in Mice With Pancreatic Cancer

**Figure 3:**
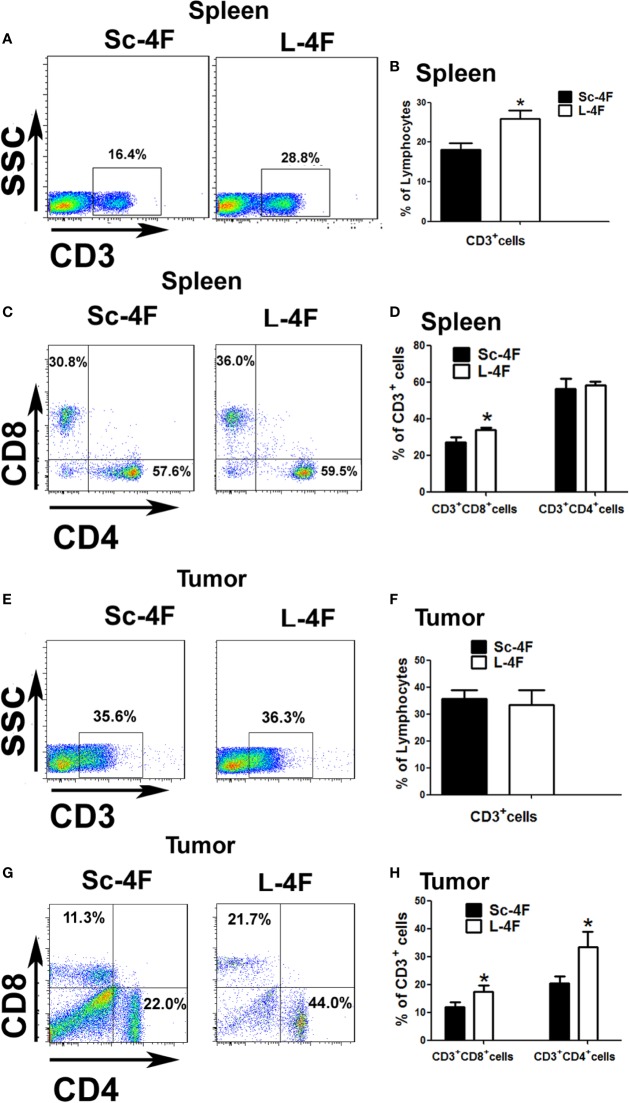
The percentages of infiltrated T cells in the spleen or tumor tissue of mice with pancreatic cancer. The spleen and tumor were collected from the Sc-4F/L-4F-treated mice. Single-cell suspensions were generated, and the cells were immunostained for CD3, CD4, and CD8. **(A, C, E, G)** One representative result from each experiment. The percentage of CD3^+^ cells among the splenocytes **(B)** and tumor-infiltrating cells **(F)** (*P < 0.05). The percentage of CD3^+^CD8^+^T cells and CD3^+^CD4^+^T cells among the CD3^+^T population of the splenocytes **(D)** and tumor-infiltrating cells **(H)** (*P < 0.05).

MDSCs have the ability to significantly inhibit immune cell response. So we detected the infiltration of T cell and T subpopulation in spleen and tumor-infiltrating cell. As shown in **Figures 3A, B**, compared with the Sc-4F treated groups, the L-4F obviously increased T cell infiltration in the spleen of treated mice (18.03% *vs* 25.9%, respectively, P < 0.05). Therefore, we further analyzed the percentages of CD3^+^CD4^+^ cells and CD3^+^CD8^+^ cells in total T cells from the spleen and the tumor infiltrating lymphocytes. In the L-4F group, the percentages of CD3^+^CD8^+^ T cells (27% *vs* 33.7%, respectively, P < 0.05) significantly increased in the spleen (**Figures 3C, D**), and the percentages of CD3^+^CD4^+^ T cells (20.4% *vs* 33.34%, respectively, P < 0.05) and CD3^+^CD8^+^ T cells (11.91% *vs* 17.41%, respectively, P < 0.05) all significantly increased in the tumor-infiltrating cell populations (**Figures 3G, H**). However, the percentages of CD3^+^CD4^+^ T cells in the spleen (56.27% *vs* 58.1%, respectively, NS) (**Figures 3C, D**) and the percentages of total T cells in the tumor-infiltrating cell populations (36.73% *vs* 33.67%, respectively, NS) did not significant changes (**Figures 3E, F**).

### L-4F Blocks the Immunosuppressive Function of MDSCs

**Figure 4:**
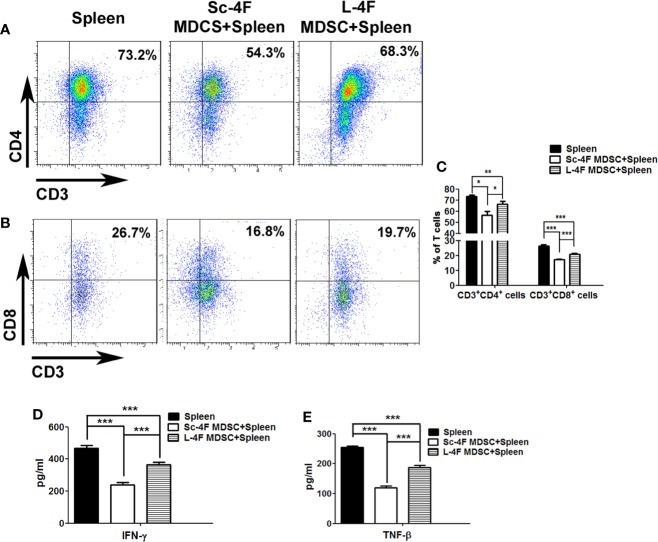
L-4F blocks the immunosuppressive function of MDSCs Ly6G^+^Ly6C^+^ MDSCs were isolated from the Sc-4F- or L-4F-treated mice and co-cultured with splenocytes from normal mice. **(A, B)** One representative result from each experiment is shown. **(C)** The percentages of CD3^+^CD4^+^ T cells and CD3^+^CD8^+^ T cells in T cell population (***P < 0.001,**P < 0.01, *P < 0.05). **(D)** The concentration of IFN-γ in the culture medium (***P < 0.001). **(E)** The concentration of TNF-β in the culture medium (***P < 0.001).

To detect the immune suppression mediated by MDSCs, Ly6G^+^Ly6C^+^ MDSCs isolated from the Sc-4F or L-4F treated mice were co-cultured with splenocytes from normal mice. Then we analyzed the percentages of CD3^+^CD4^+^ T cells and CD3^+^CD8^+^ T cells in the total T cell population. Compared with the without co-cultured MDSCs group, CD3^+^CD4^+^ T cells percentage (73.35% *vs* 56.57%, respectively, P < 0.05) and CD3^+^CD8^+^ T cells percentage (26.53% *vs* 17.57%, respectively, P < 0.01) significantly decreased in co-cultured with MDSCs which from Sc-4F treatment group. While, compared with the co-cultured with the MDSCs which from Sc-4F treatment group, the percentages of CD3^+^CD4^+^ T cells (56.57% *vs* 66.42%, respectively, P < 0.05) and CD3^+^CD8^+^ T cells (17.57% *vs* 20.85%, respectively, P < 0.01) (**Figures 4A–C**) increased in co-cultured with MDSCs which from the L-4F-treated group.

The concentration of IFN-γ and TNF-β from the culture medium was detected after co-culturing MDSCs with T cells as performed for the immune-suppression assays. Compared with the without MDSCs group, the concentrate of IFN-γ from the co-culturing with MDSCs which from Sc-4F treatment group obviously decreased (468.33 pg/ml *vs* 237.85 pg/ml, P < 0.01). While compared with the co-culturing with MDSCs which from Sc-4F treatment group, the concentrate of IFN-γ from the co-culturing with MDSCs which from L-4F treatment group obviously increased (237.85 pg/ml *vs* 364.98 pg/ml, P < 0.01) (**Figure 4D**). Similar, compared with the without MDSCs group, the concentrate of TNF-β from the co-culturing with MDSCs which from Sc-4F treatment group obviously decreased (254.21 pg/ml *vs* 119.28 pg/ml, P < 0.01). While compared with the co-culturing with MDSCs which from Sc-4F treatment group, the concentrate of TNF-β from the co-culturing with MDSCs which from L-4F treatment group obviously increased (119.28 pg/ml *vs* 189.38 pg/ml, P < 0.01) (**Figure 4E**).

### L-4F Inhibits the Production of ROS and H_2_O_2_ by MDSCs

**Figure 5:**
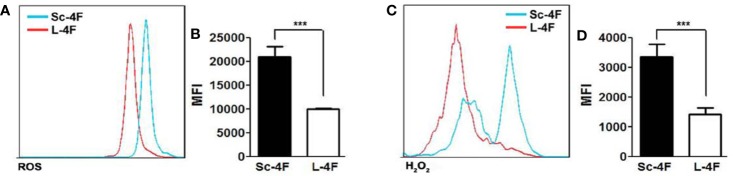
L-4F inhibits the production of ROS by MDSCs MDSCs were isolated from the spleen and tumor tissue of Sc-4F or L-4F-treated mice using anti-mouse Ly-6G and Ly-6C particles-DM. The ROS activity and H_2_O_2_ concentration were detected. **(A, C)** One representative result from each experiment is shown. The MFI of the ROS activity **(B)** and the MFI of the H_2_O_2_ detection **(D)** in the isolated Ly6G^+^Ly6C^+^ MDSCs (***P < 0.001).

It has been reported that PMN-MDSCs inhibit T cell functions *via* multiple pathways, including the up-regulation of ROS and H_2_O_2_ production and the generation of arginase-1 (Arg-1). We thus detected the ROS activity and the concentration of H_2_O_2_ in Ly6G^+^Ly6C^+^ MDSCs isolated as described in the *Materials and Methods* section. Compared with the Sc-4F-treated group, the L-4F-treated group showed significant decreases in ROS activity (MFI: 2.09×10^4^ *vs* 0.99×10^4^, respectively, P < 0.01) (**Figures 5A, B**) and the concentration of H_2_O_2_ (MFI: 3.35×10^3^ *vs* 1.41×10^3^, respectively, P < 0.01) (**Figures 5C, D**).

### L-4F Downregulates STAT3 Signaling Pathways in PMN-MDSCs

**Figure 6:**
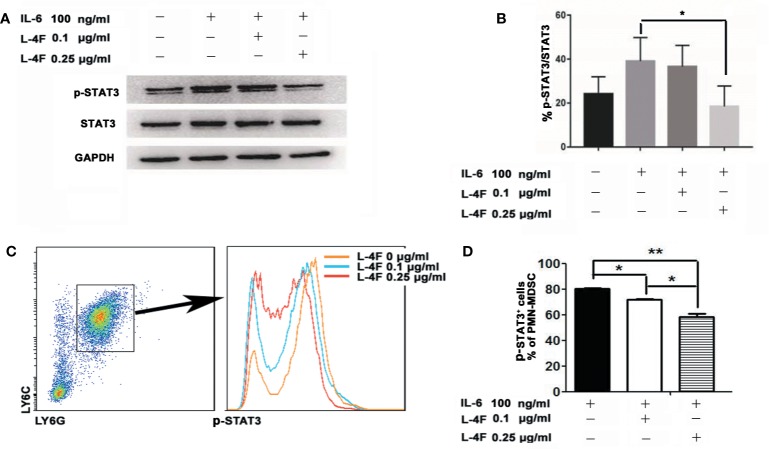
L-4F downregulates STAT3 signaling pathways in PMN-MDSCs Mouse MDSCs were induced *in vitro*. On the fourth day, the cells were incubated with L-4F at a concentration of 0, 0.1, and 0.25 μg/ml for 12 h, and then stimulated with concentration of 100 ng/ml recombinant mouse IL-6 for 15 min. **(A)** One representative result from each experiment is shown. **(B)** The ratio of p-STAT3/STAT3 in MDSCs (*P < 0.05). MDSCs were isolated from the spleen and tumor tissue from pancreatic cancer mouse model using anti-mouse Ly-6G and Ly-6C particles-DM. The MDSCs were incubated with L-4F at a concentration of 0, 0.1, and 0.25 μg/ml for 12 h, then stimulated with concentration of 100 ng/ml recombinant mouse IL-6 for 15 min. **(C)** One representative result from each experiment is shown. **(D)** The percentage of p-STAT3 in PMN-MDSCs (**P < 0.01, *P < 0.05).

To explore the mechanism by which L-4F inhibits the differentiation and immunosuppressive function of PMN-MDSCs, we first observed the effect of L-4F in the level of p-STAT3 in MDSCs by western blotting, our results show that the expression of p-STAT3 was significantly reduced after 0.25 µg/ml L-4F intervention compared with IL-6 stimulation alone (**Figures 6A, B**). Further we detected the percentage of p-STAT3 in PMN-MDSCs derived from MDSCs isolated as described in the *Materials and Methods* section. As shown in **Figures 6C, D**, L-4F obviously decreased the phosphorylation level of STAT3 (80.3% *vs* 71.73% *vs* 58.47%, P< 0.01) in a dose-dependent manner in PMN-MDSCs (**Figures 6C, D**).

## Discussion

One of the challenges of developing effective immunotherapies for clinical practice remains the complex interaction between the host immune system and the tumor, which includes the various mechanisms by which the tumor evades the immune system. A variety of cells are known to be involved in tumor-mediated immune suppression, including TAMs, regulatory T cells (Tregs), type 2 natural killer T cells, and MDSCs (Mundy-Bosse et al., 2011; Diaz-Montero et al., 2014).

Our previous results show that L-4F could inhibit pancreatic cancer progression, mostly by its anti-inflammatory effect, such as reduce the secretion of IL-6 in tumor tissue (Peng et al., 2017), While IL-6 could increase the infiltration of MDSCs in tumor tissue (Oh et al., 2013). In addition, our previous results show that reducing the infiltration of MDSCs and Treg in tumor tissue can inhibit the development of pancreatic cancer in mice (Peng et al., 2014). In this study, we elucidated the effect of L-4F on MDSCs in mouse pancreatic cancer model. Our results (**Figure 1**) show that L-4F attenuated the progression of pancreatic tumors in mice. And L-4F decreased the infiltration of PMN-MDSCs in mouse spleen and tumor tissue. Furthermore, L-4F could inhibit the differentiation of PMN-MDSCs *in vitro* (**Figures 2A, B**). However, L-4F had no effect on either the proliferation or apoptosis of MDSCs (**Figures 2C–F**). This means that the inhibition of pancreatic cancer progression by L-4F may be related to the decrease of PMN-MDSCs infiltration and PMN-MDSCs differentiation in the mouse spleen and pancreatic cancer tissue.

MDSCs have the ability to significantly inhibit immune cell response. There are several mechanisms by which MDSCs inhibit the function of T cells, including the production of arginase 1 (Arg1) and reactive oxygen species (ROS), nitrosylation of the T cell receptor (TCR), downregulation of CD62L expression, and sequestration of cysteine (Rodriguez et al., 2007; Corzo et al., 2009; Ostrand-Rosenberg, 2010). In terms of functional differences, PMN-MDSCs play its immunosuppressive function mainly depend on ROS and the enzyme Arg1, while MO-MDSCs mainly depend on nitric oxide synthase-2 (NOS2) and ROS. ROS is an important factor which PMN-MDSCs have the ability to inhibit T cells. By producing high levels of ROS, such as hydrogen peroxide and H_2_O_2_, MDSCs can cause T cell apoptosis. ROS can also cause the nitrosylation of T cell receptor during MDSC-T cell contact, which inhibit TCR bind to antigen, thus blocking T cell activation (Youn et al., 2008; Ostrand-Rosenberg, 2010; Solito et al., 2011; Kotsakis et al., 2012). In this study, our results (**Figure 3**) show that the L-4F treatment increased the infiltration of CD3^+^CD4^+^ T and CD3^+^CD8^+^ T cells into the mouse spleen and pancreatic cancer tissue to various degrees. In addition, L-4F could weaken the immunosuppressive effects of MDSCs on T cell proliferation and IFN-γ, TNF-β secretion (**Figure 4**). Furthermore, L-4F could inhibit the production of ROS and H_2_O_2_ by MDSCs (**Figure 5**). This means that L-4F can attenuate the progression of pancreatic cancer and may also be related to weakened immunosuppressive function in MDSCs.

It has been demonstrated that signal transducer and activator of STAT3 may promote the differentiation of MDSCs, and STAT3 is also known to be a key factor for the MDSCs suppressive effect. Increased ROS production in MDSCs is relate to the enhancive expression of Nox2. The activation of STAT3 directly increasing the transcription of Nox2 (Condamine and Gabrilovich, 2011). Our previous results show L-4F could prevent the differentiation of M2 macrophage, and it related to the inhibition of STAT3 pathways (Peng et al., 2017). In this study, our results (**Figure 6**) show that L-4F significantly decreased the phosphorylation of STAT3 in PMN-MDSCs in a dose-dependent manner. These results indicate that the decrease in PMN-MDSCs infiltration, inhibition of PMN-MDSCs differentiation, and weakened immunosuppressive function of PMN-MDSCs after the L-4F treatment is mediated by decreasing STAT3 phosphorylation in the tumor tissue.

## Conclusion

In conclusion, we have proven that L-4F could inhibit pancreatic cancer progression, mostly by reducing the infiltration of PMN-MDSCs and weakening their immunosuppressive function by decreasing the phosphorylation of STAT3 in the tumor tissue. Therefore, L-4F plays a crucial role in regulating tumor microenvironment. Our results indicate that L-4F represents a novel immunomodulatory candidate for the clinical treatment of pancreatic cancer.

## Data Availability Statement

All datasets generated for this study are included in the article/supplementary material.

## Ethics Statement

The animal study was reviewed and approved by medical ethics committee of Weifang Medical University.

## Author Contributions

MP, Conceptualization, Writing–original draft preparation, Investigation, Project administration. QZ, Methodology, Validation Investigation. YL, Methodology, Validation Investigation. XG, Resources, Data Curation. JJ, Funding acquisition. LX, Funding acquisition. YG, Funding acquisition. DC, Funding acquisition. DM, Writing–review and editing, Project administration. RZ, Project administration.

## Funding

This work was supported by the National Natural Science Foundation of China through grant No. 81502469, 81972695, 81602496, 81701590, and 31600775; the Science Foundation of Shandong Province through grant No. ZR2015HL061, ZR2018MH014, and ZR2017BH071; and the Natural Science Foundation of Tianjin through grant No. 18JCQNJC11100; and the foundation for visiting scholar abroad in Weifang Medical University, and Innovation and university promotion project of Guangdong Pharmaceutical University through No. 2017KCXTD020.

## Conflict of Interest

The authors declare that the research was conducted in the absence of any commercial or financial relationships that could be construed as a potential conflict of interest.

## Abbreviations

HDL, high-density lipoprotein; PBS, phosphate-buffered saline; GM-CSF, granulocyte macrophage colony stimulating factor; MDSCs, myeloid-derived suppressor cell; PMN-MDSCs, granulocytic myeloid-derived suppressor cells; MO-MDSCs, monocytic myeloid-derived suppressor cells; IFN-γ, Interferon-γ; TNF-β, Tumor necrosis factor-β; Th cells, T helper cells; PDA, pancreatic ductal adenocarcinoma; TILs, tumor-infiltrating lymphocytes; STAT3, signal transducer and activator of transcription 3.
